# The Effects of Partial Replacement of Ground Granulated Blast Furnace Slag by Ground Wood Ash on Alkali-Activated Binder Systems

**DOI:** 10.3390/ma16155347

**Published:** 2023-07-29

**Authors:** Ece Ezgi Teker Ercan, Andrzej Cwirzen, Karin Habermehl-Cwirzen

**Affiliations:** Building Materials, Department of Civil, Environmental and Natural Resources Engineering, Luleå University of Technology, 97187 Luleå, Sweden; andrzej.cwirzen@ltu.se (A.C.); karin.habermehl-cwirzen@ltu.se (K.H.-C.)

**Keywords:** wood ash, wood fly ash, ground granulated blast furnace slag, alkali-activated, mortar, ball mill, grinding, isothermal calorimetry

## Abstract

Cement production contributes significantly to carbon dioxide emissions. Alkali-activated materials offer an environmentally friendly alternative due to their comparable strength, durability and low-carbon emissions while utilizing wastes and industrial by-products. Wood ash is a waste material that shows promising results as a partial replacement for Portland cement and precursors in alkali-activated systems. The aim of this study was to examine the effect of ground wood ash on the mechanical properties of alkali-activated mortars. Wood ash was incorporated as a 0 wt%, 10 wt% and 20 wt% partial replacement for ground granulated blast furnace slag (GGBFS). The wood ashes were ground in a planetary ball mill for 10 and 20 min. Sodium silicate (Na_2_SiO_3_), sodium carbonate (Na_2_CO_3_), and sodium hydroxide (NaOH) were used as alkali activators. The results demonstrated that ground wood ash improved the mechanical properties of alkali-activated systems compared to untreated wood ash. However, the incorporation of wood ash increased the porosity of the binder matrix.

## 1. Introduction

Concrete is extensively used in the construction industry worldwide, due to its favorable attributes such as durability, mechanical properties, and affordability [1,2,3]. Unfortunately, Portland cement, which is the main concrete component, has a significant carbon footprint. Its production requires substantial amounts of raw materials and energy, leading to the release of large amounts of CO_2_, thus negatively contributing to greenhouse gas emissions and causing environmental concerns. The cement industry is estimated to produce around 5–8% of global carbon dioxide emissions [4,5]. Depending on the energy sources used, the emissions from Portland cement production range between 500 and 900 kg CO_2_/t [6,7]. Portland cement is primarily made from limestone and clay. This, involves calcination, which decomposes CaCO_3_ into CaO and CO_2_, emitting substantial amounts of greenhouse gases [8,9,10].

Alkali-activated binders, which are an eco-alternative to Portland cement, are formed through a chemical reaction between an aluminosilicate precursor and alkali activators. In systems containing high-calcium content, the main reaction product is the formation of C-A-S-H (calcium(alumino)silicate hydrate) gel, which is responsible for strength development [11,12]. Alkali-activated materials have emerged as a viable alternative to traditional Portland cement-based systems, primarily due to their significant reduction of the CO_2_ emissions, reaching up to 80% [13,14,15]. Energy consumption is lowered by 43%, and the water usage is reduced by about 25% [16,17]. Moreover, they exhibit enhanced strength, and certain durability characteristics prove superior [18,19]. In addition to all these advantages, it also provides advantages in terms of utilization of industrial waste materials and by-products such as slag or fly ash [20]. One of the most used industrial by-products as an alkali-activated precursor is ground granulated blast furnace slag (GGBFS), which comes from steel production. Alkali-activated slag systems exhibit high mechanical strength; however, they are accompanied by challenges such as rapid setting times and high shrinkage. To address these properties, their utilization in combination with other precursors has been investigated, yielding promising improvements. The combination of slag and fly ash is the most studied binary system [18]. Recently, extended setting time [21], decreased shrinkage [22], and ultra-high strength are reported for this binary system [23].

Wood ash is often considered an alternative to coal fly ash [24,25]. Wood ash is the residue produced through the incineration of forestry residues and by-product from timber manufacturing industry such as timber and sawdust [4,26]. It consists of inorganic and organic matter that remains after combustion. On average, wood burning yields approximately 6–10% ash [27]. Wood biomass is considered CO_2_ neutral energy source which emits almost the same amount of CO_2_ when burnt as it absorbs during its growth [28,29,30]. Globally, the annual production of woody biomass is approximately 4600 million tons. Out of this total, 60% is utilized for energy production, 20% is used for industrial purposes, and 20% is lost during primary production and decomposes in the field [31]. Nowadays, a significant amount of wood ash is disposed of in landfills, although some are employed in agriculture and forestry [24,32]. However, there are concerns associated with these applications of wood ash. Firstly, future difficulties in finding suitable landfill areas are anticipated, leading to increased costs [10,33]. Furthermore, landfilling of wood ash can result in the leaching of hazardous elements, potentially contaminating groundwater [34]. Health risks may also arise from the disposal of wood ash in landfills, as fine particles can become airborne and disperse through wind [24,35]. The presence of heavy metal content and acidic pH levels of wood ash may pose potential risks in some agricultural practices [36]. Compared to other disposal methods of wood ash, incorporating it into concrete might be a more sustainable alternative [27].

The composition of wood ash varies based on geographical location, tree species, parts of the tree, combustion technology and temperature, as well as collection methods and storage conditions [32,37,38]. It usually contains a high amount of CaO and SiO_2_ [39].

Studies on the use of wood ash in environmentally friendly construction materials are increasing [29,35,38,40] and these studies were summarized in [7]. The utilization of wood ash as a partial Portland cement replacement has emerged as a promising application for mitigating the carbon footprint of the industry, at least for some applications. This approach not only helps to reduce cement consumption but also offers a sustainable way for waste disposal [41]. Compared to Portland cement, wood ash has larger, irregular, and more porous particles with a larger specific surface. Incorporation of wood ash usually decreases workability [9,34,38,42] of cement-based materials and results in slightly lower or improved mechanical properties [41,43,44]. A Portland cement replacement by 10–20 wt% with wood ash was suggested as optimum [34,43,45,46].

In addition to the utilization of wood ash as a partial cement replacement, there has been a growing research focus on the application of wood ash in the field of alkali-activated materials. Some researchers proposed using wood ash as an alkaline source for alkali-activated materials without using any chemical alkali solution due to the potential negative environmental impacts of sodium silicate and sodium hydroxide. Cheah et al. [47] successfully replaced fly ash with higher amount of wood ash, 50, 60, 70 and 100 wt% without using any alkali-activator. They achieved the maximum compressive strength of 18 MPa with 60 wt% wood ash at the age of 90 days. The high K_2_O and CaO content in the wood ash makes it suitable for geopolymerization. The formation of K-A-S-H, C-A-S-H, and C-S-H was observed as a result of the reaction with water. Samsudin and Cheah [48] examined high-calcium wood ash as a substitute for ground granulated blast furnace slag (GGBFS) in geopolymer concrete without a chemical alkali activator. A replacement ratio of 30% yielded the highest compressive strength at 12.3 MPa with a rapid early-stage strength development due to the high alkalinity of wood ash.

In addition to using wood ash as an alkaline source, it can be also used as a partial replacement for fly ash, metakaolin and GGBFS. Owaid et al. [20] suggested using up to 25 wt% wood ash as a partial replacement for fly ash in geopolymer concrete to enhance its cost-effectiveness and environmental sustainability. Different wood ash ratios were compared (25, 50, 75, and 100 wt%) using sodium silicate and sodium hydroxide as activators. Curing involved 24 h at 60 °C followed by an ambient temperature curing. The geopolymer concrete with 25 wt% wood ash replacement exhibited the highest compressive strength (57.82 MPa on the 56th day), while higher wood ash ratios led to decreased mechanical properties, presumably due to high CaO content in wood ash. Abdulkareem et al. [49] also observed that the incorporation of up to 20 wt% of wood ash as a partial replacement for fly ash in geopolymer mortars shortened initial and final setting times and improved compressive strength at early ages due to the formation of C-S-H and geopolymer gels. Cheah et al. [50] investigated the replacement of fly ash with high-calcium wood ash at various ratios in geopolymer mortars. They used a sodium silicate solution as an alkali activator with an alkali modulus of 2.1. The study found that the highest compressive and flexural strength was achieved with 40 wt% and 50 wt% wood ash replacements at 7 and 28 days. Interestingly, the samples containing 30 wt% wood ash exhibited the highest mechanical strength after 365 days. Moreover, as the wood ash content increased in the binder, the amount of mixing water required to achieve the standard consistency also increased.

Candamano et al. [51] examined the effect of wood ash as a partial replacement for metakaolin in geopolymer mortars. The incorporation of 10–30 wt% wood ash improved the workability, but when more than 10 wt% of wood ash was used, there was a decrease in compressive and flexural strengths. However, even with a 30 wt% replacement ratio, the strength remained above 35 MPa, demonstrating the potential of wood ash in geopolymer applications. De Rossi et al. [52] investigated the effects of different curing methods on the properties of geopolymer mortars using 75 wt% wood fly ash and 25 wt% metakaolin. The study used two ratios of sodium silicate to sodium hydroxide and subjected the samples to five curing methods. The results showed that a higher sodium silicate to sodium hydroxide ration improved the compressive strength and reduced the water absorption due to the higher SiO_2_/Al_2_O_3_ ratio for all the curing methods except hydrothermal curing. The highest compressive strength of 24.5 MPa was observed in geopolymers cured at 40 °C, but this method was not suggested due to increased carbon dioxide emissions and energy consumption. Room temperature curing was found to be a cost-effective and environmentally friendly alternative, resulting in the formation of geopolymers with similar mechanical properties.

Silva et al. [53] investigated the use of untreated wood ash as a precursor with NaOH solution with varying concentrations used as an alkali activator. The mixes with 100 wt% wood ash showed porous and heterogeneous structure with microcracks due to drying during curing and low-compressive and flexural strength due to the coarse wood ash particles. The authors suggested sieving and crushing processes as pre-treatments for wood ash to achieve improved mechanical properties. Bajare et al. [54] conducted a study where wood ash that was used as a precursor had been ground with a planetary ball mill for 10 min to enhance its reactivity. They used 6 M NaOH solution as an alkali activator. The geopolymer mortars cured at 75 °C for 24 h reached a compressive strength of 9.3 MPa. Ates et al. [55] investigated the impact of calcination and ball milling on the compressive and flexural strength of fly ash-wood ash blended geopolymer mortars. They observed that these processes improved the strength properties of the mortars, up to a 50 wt% wood ash level.

Wood ash has high unburned carbon content, which decreases its reactivity. To enhance the reactivity of wood ash, different pre-treatment methods such as sieving, grinding, calcination and water-washing have been suggested [28,38,56,57]. Besides reducing loss on ignition, pre-treatments like grinding and water-washing can improve the physical and chemical properties of wood ash. Grinding also enhances particle reactivity by reducing particle size. These treatments can potentially impact the strength and durability of concrete [58].

Researchers have explored the potential of utilizing wood ash as a partial replacement for cement, alkali-activated fly ash or metakaolin to some extent. However, not enough studies examine its usage with slag. This study used wood ash as 10 wt% and 20 wt% partial slag replacement in alkali-activated mortars after grinding wood ash with two different grinding times, 10 or 20 min. Mortars were prepared with three different alkali activators, namely sodium silicate (Na_2_SiO_3_), sodium carbonate (Na_2_CO_3_) and sodium hydroxide (NaOH). The aim was to examine the effect of ground wood ash at various times on the mechanical properties of alkali-activated mortars with different alkali-activator types.

## 2. Materials and Methods

### 2.1. Materials

**Figure 1:**
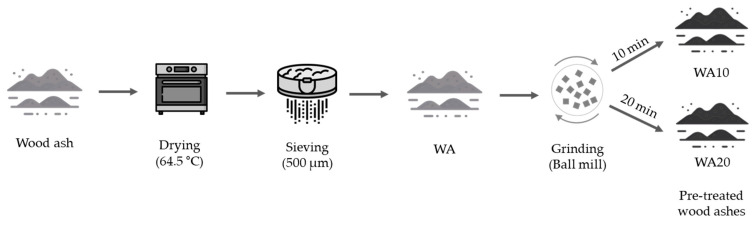
Processing of wood ash after receiving.

The wood fly ash (WA) utilized in this study was sourced from Stenvalls Trä AB (Pitea, Sweden). It was a by-product of wood waste combustion. The wood ash was dried in the oven at 64.5 °C for 24 h and sieved with a 500 μm sieve before grinding [56]. Then it was ground using a planetary ball mill at a rotation speed of 500 rpm, with a ball-to-powder ratio of 5. The grinding process was conducted for two different durations of 10 and 20 min (Figure 1). The equipment use was a Retsch PM100 planetary ball mill (Retsch GmbH, Haan, Germany), with a jar having a 500 mL capacity and 10 balls with a diameter of 20 mm. The mean particle sizes of untreated and treated wood ashes were measured based on SEM images by using ImageJ software (v.2.14.0). Mean particle sizes of untreated wood ash (WA), 10 min of ground wood ash (WA10) and 20 min of ground wood ash (WA20) were measured at 18.31 μm, 3.79 μm and 4.22 μm, respectively. The chemical compositions and pH values of untreated WA, pre-treated WAs (WA10 and WA20), and GGBFS are provided in Table 1. The reason for the oxide losses in wood ash was attributed to the high =0sulfur content of the wood ash that was lost during burning.

Ground granulated blast furnace slag (GGBFS) was obtained from SweCem (Helsingborg, Sweden). The basicity coefficient (Kb) of GGBFS was found to be 1.13, which is greater than 1, indicating its basic nature [59]. The pH value of GGBFS was measured to equal 10.38. The calculation was performed using the equation Kb = (CaO + MgO)/(SiO_2_ + Al_2_O_3_) [15,60,61]. Furthermore, the hydration modulus (HM) of the slag is determined to be 1.82. The hydration modulus was calculated using the equation HM = (CaO + MgO + Al_2_O_3_)/SiO_2_ [60]. The HM of slag was suggested to be greater than 1.4 for ensuring efficient hydration products [60,62]. The fine sand B35 (350 µm), which was used in mortars, was provided by Baskarpsand AB (Habo, Sweden).

Three alkali activators were used: sodium silicate, sodium carbonate, and sodium hydroxide. The liquid sodium silicate (SS) (Na_2_SiO_3_) was provided by Sigma-Aldrich and alkali modulus is calculated as Ms = SiO_2_/Na_2_O of 2.5 with 26.5 wt% SiO_2_, 10.6 wt% Na_2_O, and a solid content of 43.82 wt%. The Ms value was adjusted to 1 by adding sodium hydroxide (NaOH) pellets (98% purity), which was provided by Sigma-Aldrich. The alkali activator dosage was 10 wt% for all SS-activated mixes.

Sodium carbonate (SC) (Na_2_CO_3_) powder was provided by CEICH SA (Warsaw, Poland). A total of 10 wt% SC was used in all SC-activated mixes. Sodium hydroxide (SH) solution of 10 M concentration was prepared with distilled water for the SH-activated mixes.

Mortar samples were used for compressive and flexural strength measurements, and to perform SEM and EDS spot analysis. A total of 21 different alkali-activated mortar mixes were prepared with GGBFS, replaced by 0 wt%, 10 wt%, and 20 wt% of untreated wood ash (WA); 10 min of ground wood ash (WA10); and 20 min of ground wood ash (WA20). The mix design of the alkali-activated mortars is provided in Table 2. The first number after the alkali-activator code in the mortar ID represents the wood ash ratio in the mix and the second number represents wood ash grinding duration. The control mortar samples were prepared using 100 wt% GGBFS for all three alkali-activator types. The water-to-binder (w/b) ratio was 0.5 and the sand to binder (s/b) was 2 for all mortar mixes. SS and SH solutions were prepared approximately 3 h before casting. Firstly, dry materials were mixed in a Hobart mixer for three minutes, and then alkali activators dissolved in water were added and mixed for another two minutes. The samples were cast into the molds with a dimension of 40 × 40 × 160 mm^3^, then sealed with plastic foil and stored at ambient conditions. After demolding, all samples were kept sealed in plastic bags.

The paste samples were prepared for XRD analysis and isothermal calorimetry measurements. Pastes were mixed for 2 min in a small volume vacuum mixer, type Ecovac (Bredent/Senden, Germany) at a mixing speed of 600 rpm.

### 2.2. Testing Methods

The X-ray diffraction (XRD) analysis was performed on powdered samples and 7 days old pastes of selected mixes by using type Empyrean from PANalytical with PIXcel 3D detector, and at operating conditions of 45 kV and 40 mA, Cu-K radiation with a wavelength of 1.54 Å, the step size was 0.02 with the range of angle 2θ from 5° to 70° [63]. The Crystallography Open Database (COD) was used to determine phase composition.

The workability of fresh mortars was determined by flow test according to ASTM C 1437-07 [64].

The microstructure and morphology of the wood ashes and hardened alkali-activated mortar samples were examined using a scanning electron microscope (SEM), JSM-IT100 (JEOL Ltd., Tokyo, Japan) connected with an energy dispersive spectrometer, BRUKER (JEOL Nordic AB, Sollentuna, Sweden), and the ESPRIT software (v.2.1).

The powder samples were placed on adhesive carbon tape. No additional conductive coating was applied. All images were obtained using a secondary electron detector (SED) in high vacuum at magnifications of 500×.

For the alkali-activated mortar sample preparation, mortar pieces were extracted from the core of 28-day-old samples and were kept in isopropanol for 48 h to stop ongoing reactions. Then the samples were stored in a desiccator for 48 h. In the next step, the samples were impregnated with a Struers EpoFix low-viscosity epoxy resin under vacuum. After the resin had hardened, the samples were polished with grinding plates covered with a diamond spray having particle sizes of 9, 3, and 1 μm. The lamp oil was used as a lubricant [65]. The SEM analysis was performed using a backscattered electron detector (BSE) in low vacuum mode. The accelerating voltage was 15.0 kV and the probe current of 50 mA at 1000× magnification. EDS spot analysis was performed at a magnification of 1000× by choosing 100 points manually based on the grey level histogram that corresponded to C-S-H [65].

The approximate porosity distribution was estimated by analyzing SEM images with the ImageJ analysis software [66]. The images were captured at multiple locations using a magnification of 500×. A Gaussian filter was applied to all analyzed images to decrease noise levels and improve the differentiation between hydration phases and pores by enhancing their contrast [67,68]. The mean particle size was determined through the analysis of SEM images. ImageJ software was utilized for image processing and analysis. To eliminate noise, a median filter with a 2-pixel kernel was applied. Subsequently, automatic thresholding was employed to convert the images into binary format [59,69].

The compressive strength test was performed at 7 and 28 days in an area of 40 × 40 mm^2^ from both ends of each mortar beam [70].

The flexural strength was determined at 7 and 28 days following by the BS EN 196-1:1995 [71]. A Wykeham Farrance mechanical testing machine was used with 0.5 mm/min loading speed and Catman Easy software. Three samples were used for each mix for calculating the average compressive and flexural strength.

Isothermal calorimetry measurements were performed using a TAM Air isothermal calorimetry. SS, SC and SH-activated paste samples containing 20 wt% of ground wood ash for 10 min and 20 wt% of untreated wood ash, and 100 wt% slag-containing samples were selected for isothermal calorimetry measurement. In total, 8.3 g of paste samples were placed into glass ampoule and kept there for 7 days at a base temperature of 23 ± 0.02 °C [72].

## 3. Results and Discussions

**Figure 2:**
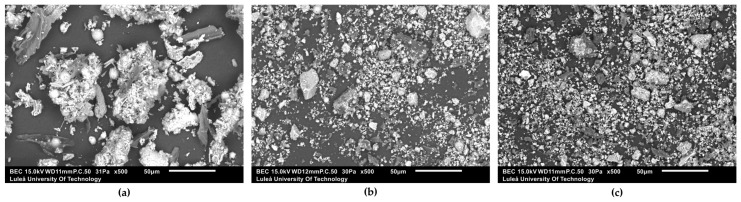
SEM images of (**a**) WA, (**b**) WA10, (**c**) WA20 at 500× magnification.

The mean particle sizes of WA, WA10 and WA20 are provided in Table 1. WA had larger and more irregularly shaped particles compared to WA10 and WA20 (Figure 2). Ball milling had a positive effect on reducing the size of the wood ash particles and led to homogenous shapes. The mean particle size of WA10 was determined to be smaller than that of WA20. This difference was attributed to the phenomenon of agglomeration, which played a significant role in influencing the particle size distribution. Milling is known to reduce particle size. However, it has been noted that milling operations beyond a critical intermediate milling time may stop or have adverse effects on particle size reduction [69,73]. Due to agglomeration, the particle size distribution widens and most of these changes occur within the first hour of milling. Kumar and Kumar [74] also stated that a significant part of the particle size reduction occurred in the first 10 min of the fly ash grinding process and then this reduction effect decreased. Besides agglomeration, the morphology of the particles becomes more irregular compared to shorter grinding durations as a result of long grinding processes [73]. Moreover, smaller particles in the grinding media can lead to coverage of grinding balls and subsequently reduce the efficiency of the milling process in time [75]. Additionally, the wear out of the grinding media represents another significant factor that influences the grinding kinetics and reduces the overall milling efficiency [76].

**Figure 3:**
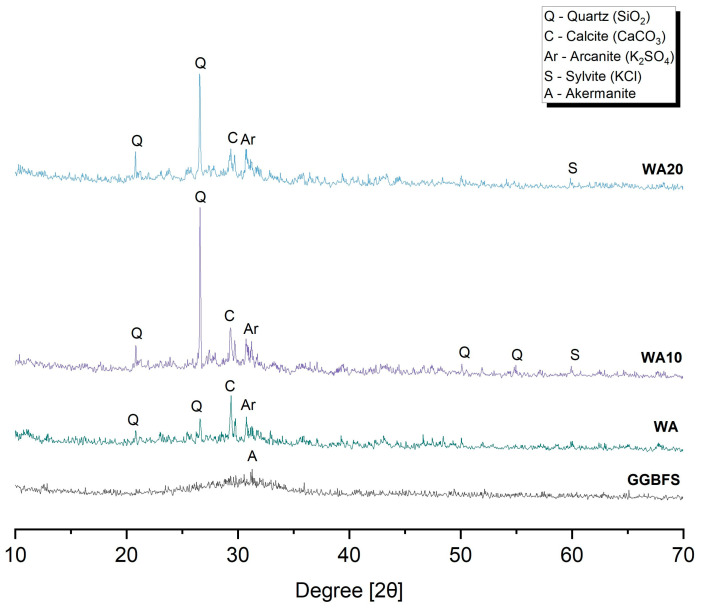
XRD patterns of WA, WA10, WA20, and GGBFS.

The XRD patterns for WA, WA10, WA20 and GGBFS are shown in Figure 3. For ground wood ashes, WA10 and WA20, the main peak was quartz, which exhibited relatively high intensities. These findings align with the literature, as many researchers have reported that quartz is the predominant crystalline phase identified in wood ash [24,44,53,63,77,78,79]. Additionally, they contained calcite, arcanite, and sylvite. In contrast, the XRD pattern of untreated wood ash, WA, exhibited the main peak corresponding to calcite, while quartz peaks were observed with lower intensities and arcanite was also detected. On the other hand, GGBFS contains akermanite as the primary phase.

### 3.1. Workability

**Figure 4:**
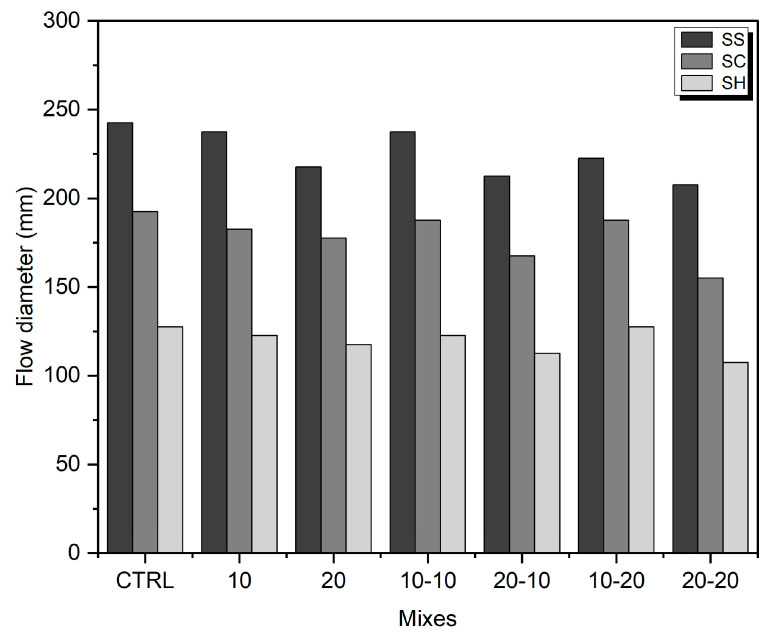
Flow diameters of the fresh mortar mixes.

The measured flow diameter values for various mixtures are shown in Figure 4. It was observed that the workability of SS-activated samples was higher than those activated with SC and SH. Notably, the control samples exhibited the highest flowability with the ranking order of SS > SC > SH. The flowability decreased with a higher amount of WA and longer grinding times. This trend in workability remained consistent across different alkali-activator types.

### 3.2. Mechanical Properties

**Figure 5:**
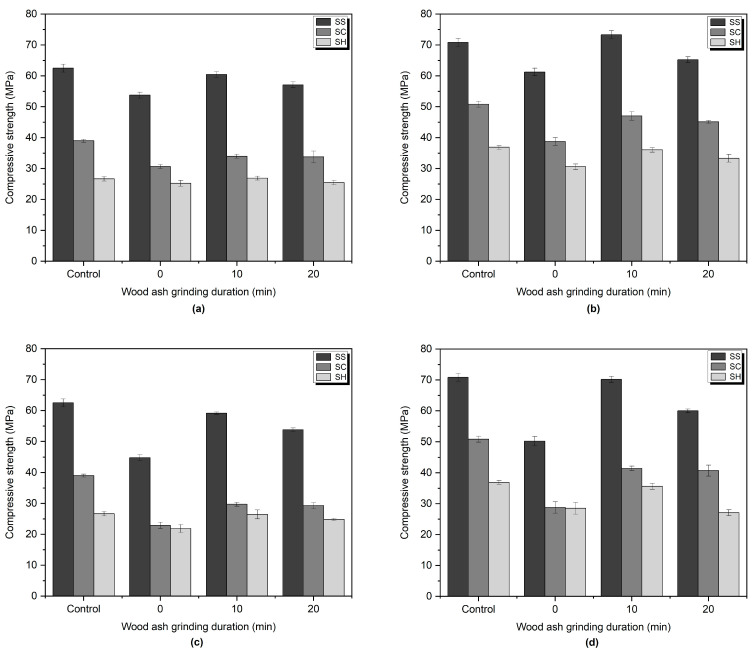
Compressive strength of SS, SC, and SH-activated mortars containing (**a**) 10 wt% wood ash at 7 days, (**b**) 10 wt% wood ash at 28 days, (**c**) 20 wt% wood ash at 7 days, (**d**) 20 wt% wood ash at 28 days.

The compressive strength results are presented in Figure 5. Mixes activated with SS displayed the highest compressive strength values at both 7 and 28 days. The incorporation of untreated wood ash reduced the compressive strength compared to the control sample. This is attributed to the high unburned carbon content, and larger and unreactive wood ash particles [63,80]. Regardless of the alkali-activator and the used grinding time of wood ash, it was observed that the compressive strength decreased with the increasing wood ash ratio. However, the results indicated that grinding had a significant impact on the compressive strength of all used alkali activator types due to increased reactivity of wood ash particles [56,81,82,83].

The 28 days old SS-activated samples containing 10 wt% wood ash and subjected to 10 min of grinding exhibited a 19.72% increase in compressive strength, while those ground for 20 min showed a 6.45% increase. The same trends were observed for SC and SH-activated samples, where grinding for 10 and 20 min led to compressive strength increases of 21.39% and 16.44%, and 17.82% and 8.96%, respectively.

Furthermore, the grinding process showed significantly greater improvements in compressive strength in samples incorporating 20 wt% wood ash. SS-activated samples exhibited increases of 39.81% and 19.50% after 10 and 20 min of grinding, respectively. Similarly, SC-activated samples showed increases of 43.97% and 41.40%, while SH-activated samples displayed increases of 32.52% and 11.51% for the same grinding durations. Specifically, 10 min of grinding had a stronger impact on the compressive strength.

In the case of SS-activated samples with a 10 wt% wood ash ratio and ground for 10 min, (SS-10-10) reached 73.33 MPa at 28 days and a slight increase of 3.5% was observed compared to the control sample. Samsudin et al. [48] attributed that the incorporation of wood ash increased the compressive strength, because the high-alkaline content of wood ash may have promoted the dissolution of aluminosilicate materials.

The strength development of the mixes was governed by the C-A-S-H gel as the Ca/Al achieved a ratio greater than 2 [84]. SS-activated and SC-activated samples might have led to the formation of the sodium polysialate product due to their Na/Si ratios ranging between 0.3 and 0.7 [84]. Average Na/Si ratios of SH-activated mortars ranged between 1.15 to 3.79. Cheah et al. [84] explained the high-Na/Si ratio observed, suggesting that rather than the formation of sodium polysialate, the presence of raw sodium ions is produced by sodium hydroxide in the pore solution.

Also, the lower compressive strength of SC-activated samples at an early age might be explained by decreasing OH^−^ and increasing CO_2_^3−^ concentrations [85]. Moreover, the late strength development of SC-activated mortars might be attributed the formation of CaCO_3_, which delays the hydration, before the C-A-S-H [86].

In the literature, it was stated that the low Ca/Si ratio has a positive effect on mechanical properties [87]. However, the presence unreacted of particles and porosity significantly affect compressive strength of the mortars. Calcium in the precursors combines with silicon ions in an alkaline environment and precipitates as C-S-H gel and supports the formation of nucleation sites, which contribute to strength development [53]. Moreover, while GGBFS provided the main contribution to strength development, wood ash fillers may have shown an effect and supported C-S-H formation by acting as nucleation sites [88,89,90,91]. In addition, it might cause a dilution effect due to the decrease in GGBFS particles with the increasing amount of wood ash, allowing more space for the formation of slag hydrates, thus increasing the degree of hydration [89,92]. In particular, fine particles of ground wood ash also increase the possibility of forming nucleation sites [24,38]. In this way, the hydration reaction can be accelerated, and more hydration products can be obtained, thereby reducing the porosity [86].

**Figure 6:**
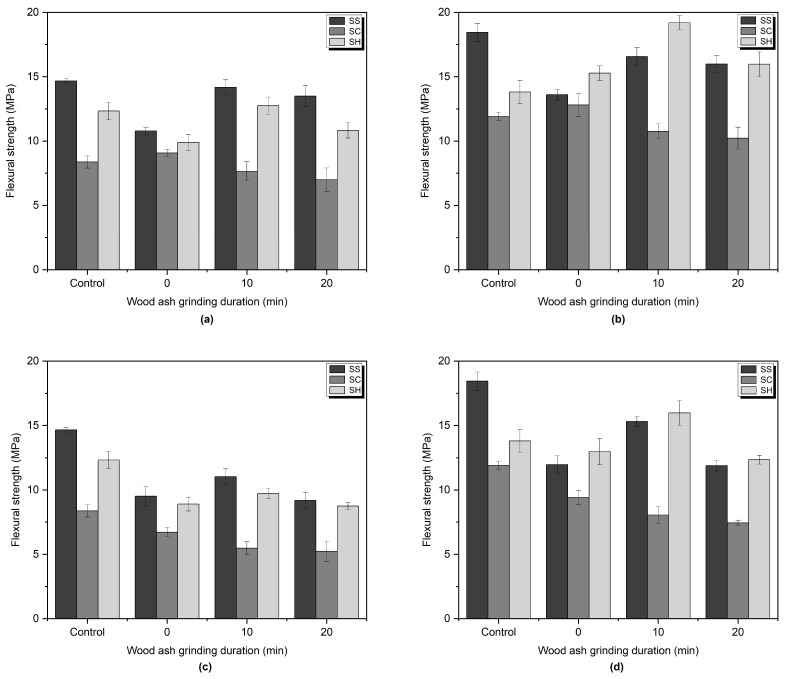
Flexural strength of SS, SC, and SH-activated mortars containing (**a**) 10 wt% wood ash at 7 days, (**b**) 10 wt% wood ash at 28 days, (**c**) 20 wt% wood ash at 7 days, (**d**) 20 wt% wood ash at 28 days.

The flexural strength results of the alkali-activated mortars at the age of 7 and 28 days are given in Figure 6. In general, the flexural strength showed a similar trend as the measured compressive strength within same alkali-activator type. However, SH-activated samples showed surprisingly high-flexural strength. Notably, all SH-activated samples incorporating 10 wt% wood ash demonstrated superior flexural strength compared to the control sample after 28 days, and SH-10-10, which contains 10 wt% of 10 min ground wood ash, achieved 19.19 MPa of flexural strength, which is the highest value among all the tested samples. The increase of the flexural strength of all SH-activated samples incorporating wood ash ranged from 41.02% to 58.89%, whereas the control sample exhibited an increase of only 11.97%. The underlying cause of this phenomenon has not been fully understood, necessitating further investigation and research.

Samples containing 20 wt% of WA ground for 10 min showed the highest flexural strength from 7th day to 28th day. The early strength development was attributed to the reactions of the slag, while the later strength gains may have been due to the effect of the wood ash [41,93].

Lower flexural strength results of SC-activated samples compared to other activator types might be attributed to higher porosity and formation of microcracks of the SC-activated samples. Aydın et al. [60] stated that flexural strength is affected by microcracks more than the compressive strength.

### 3.3. Microstructural Investigation

**Figure 7:**
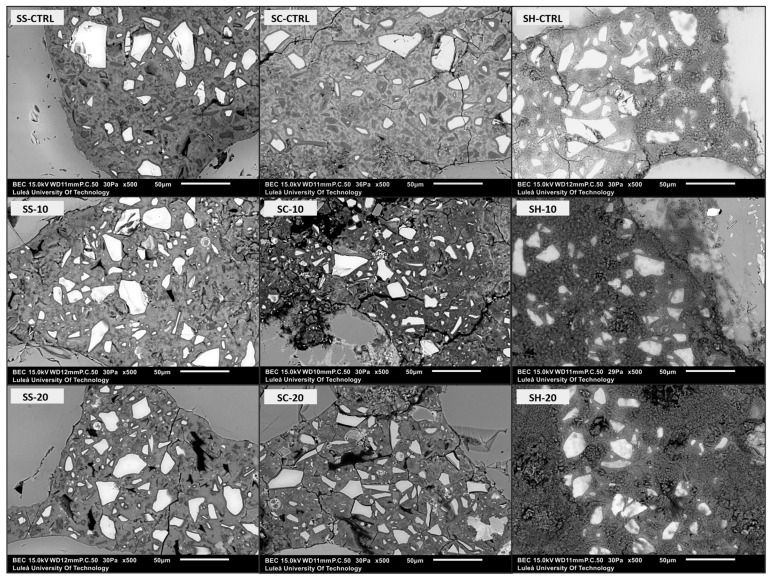
Micrographs of the 28 days old alkali-activated mortars at 500× magnification.

**Figure 8:**
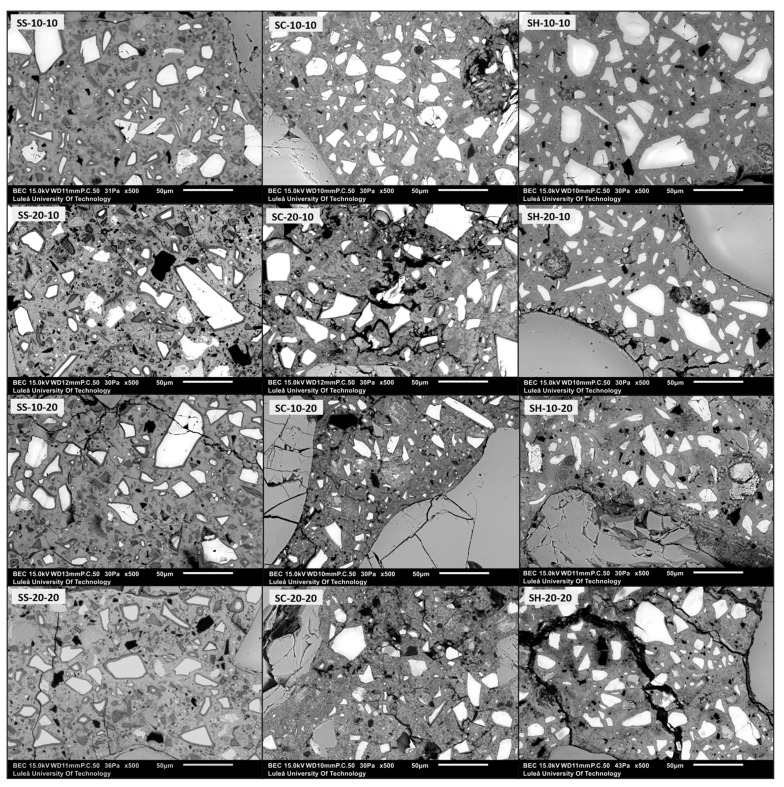
Micrographs of the 28 days old alkali-activated mortars at 500× magnification.

SEM analysis was conducted on the polished surfaces of the 28 days old alkali-activated mortar samples. The micrographs of the samples obtained at 500× magnification are presented in Figure 7 and Figure 8. Notably, the microstructure of all the control samples and those containing 10 wt% of wood ash ground for 10 min were more homogeneous and denser, irrespective of the type of alkali activator used. With an increase in the wood ash ratio and microcracks, the amount of unreacted GGBFS and wood ash increased. The presence of microcracks might affect the mechanical strength and long-term durability of the material. The formation of microcracks is attributed to drying and shrinkage [47,51,94,95]. The wood ash ground for 10 min appeared to be better dispersed within the binder matrix. On the other hand, samples containing wood ash ground for 20 min exhibited a higher amount of unreacted wood ash particles.

**Figure 9:**
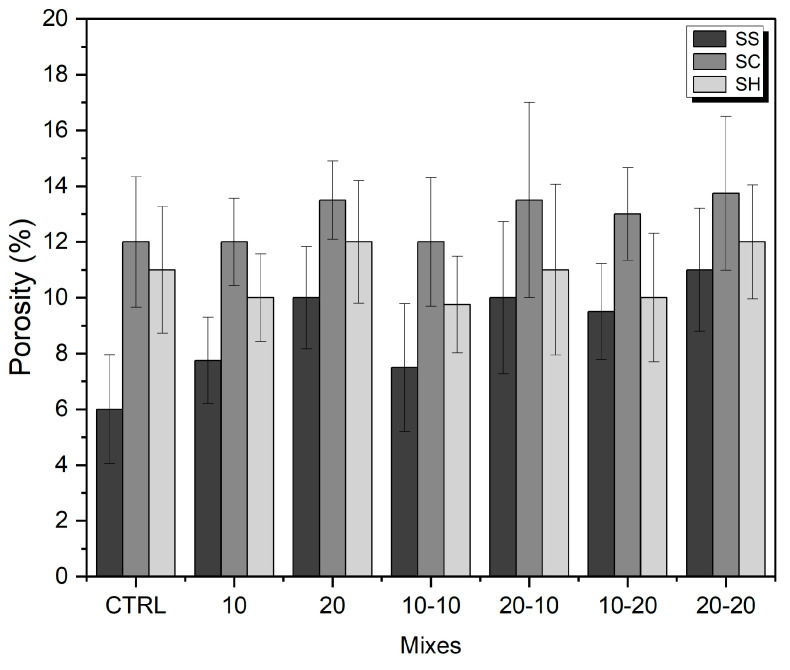
Estimated total porosity of the alkali-activated mortars.

The approximate porosity ratios of the alkali-activated mortar samples were estimated using SEM micrographs and presented in Figure 9. Consistent with the literature findings [51,55], the incorporation of wood ash caused an increase in porosity, regardless of the alkali-activator type. Moreover, increasing the wood ash ratio showed an increased porosity of the mortars, except for 10 wt% wood ash containing SH-activated samples, which showed slightly lower porosity than the control sample. This might be due to the limitations of the used image analysis technique. Hu et al. [96] attributed this effect to the addition of fly ash, which led to an increase in the porosity by reducing the content of the dense calcium-aluminum-silicate-hydrate C-A-S-H gel and promoting the formation of high-porosity sodium-aluminum-silicate-hydrate N-A-S-H gel. The mortar samples containing WA10 exhibited a slightly lower porosity compared to the samples containing WA20. This might be explained by the smaller particle size of 10 min ground wood ash (WA10) in comparison to 20 min ground wood ash (WA20), which may contribute to enhanced reactivity. This might also be supported by SEM micrographs. As the samples containing WA20 had more unreacted wood ash particles, SS-activated mortars exhibited lower porosity and a denser microstructure when compared to SC and SH-activated mortars. The lower porosity of the SS-activated samples was attributed to the Si content in the medium [19]. However, the SH-activated mortars had slightly higher porosity compared to SS-activated samples, yet lower than SC-activated samples. This observation can be attributed to the higher pH value of the SS and SH solutions, which potentially enhances the dissolution of GGBFS compared to other alkali activators. The elevated pH value promotes the formation of hydration products, resulting in a denser matrix through pore filling [86].

According to the performed EDS spot analysis, the main hydration product was C-A-S-H gel. The Ca/Si atomic ratios are provided in Table 3. Average Ca/Si atomic ratios for all mixes were found between 0.6 and 1.5, which corresponds to the C-A-S-H gel [59]. Moreover, the Ca/Si ratio of SS-activated mixes were lower compared to other activated samples. This is attributed the increasing of SiO_4_^4−^ ions that are forming more hydration products. It also helps to increase the alkali-binding capability of C-A-S-H, and to accelerate the hydration [97].

### 3.4. Phase Development

**Figure 10:**
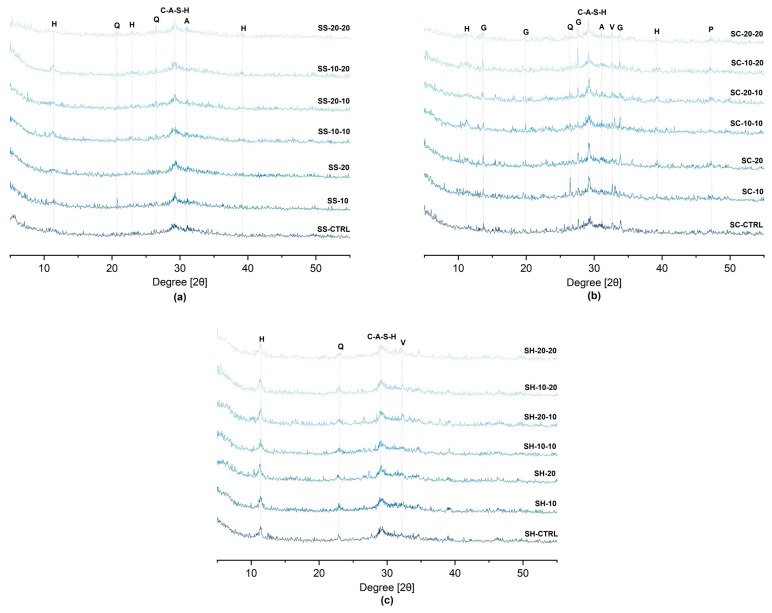
X-ray diffractograms of (**a**) SS-activated, (**b**) SC-activated, (**c**) SH-activated pastes at the age of 7 day (H—Hydrotalcite, G—Gaylussite, V—Vaterite, Q—Quartz, M—Mullite, A—Akermanite, P—Portlandite).

The XRD patterns of alkali-activated pastes, provided in Figure 10, shows the crystalline phases during the hydration. The main peak was attributed to C-A-S-H gel for the all alkali-activator types [85,86,98]. These findings supported the EDS spot analysis results.

The following crystalline phases can be observed for all alkali-activator types: hydrotalcite (Mg_6_Al_2_CO_3_(OH)_16_.4H_2_O) and quartz (SiO_2_). Additionally, akermanite (Ca_2_MgSi_2_O) in the SS-activated; gaylussite (Na_2_Ca(CO_3_)_2_.5H_2_O), portlandite (Ca(OH)_2_), mullite, vaterite and akermanite in the SC-activated samples, and vaterite for SH-activated samples were observed.

The presence of hydrotalcite and gaylussite is also consistent with the literature [85,99,100,101]. The formation of hydrotalcite mainly depends on the high-MgO content of GGBFS [99,102]. The formation of gaylussite might affect the compressive strength development of SC-activated samples [101].

### 3.5. Reaction Heat Development

In order to obtain further insights into the reactions and phase development in the alkali-activated systems, isothermal calorimetry analyses were performed on selected mixes which were SS-CTRL, SS-20, and SS-20-10; SC-CTRL, SC-20, and SC-20-10; SH-CTRL, SH-20, SH-20-10. According to the compressive strength test results, samples incorporating wood ash, that were ground for 10 min, showed higher strength values across various replacement ratios and alkali-activator types. As a consequence, the mixes containing WA10 were chosen for the calorimetry measurements. Although the highest compressive strength values were obtained with 10 wt% wood ash, a 20 wt% wood ash replacement was selected to better assess the grinding effect of wood ash. Heat development was measured for a total of 168 h.

**Figure 11:**
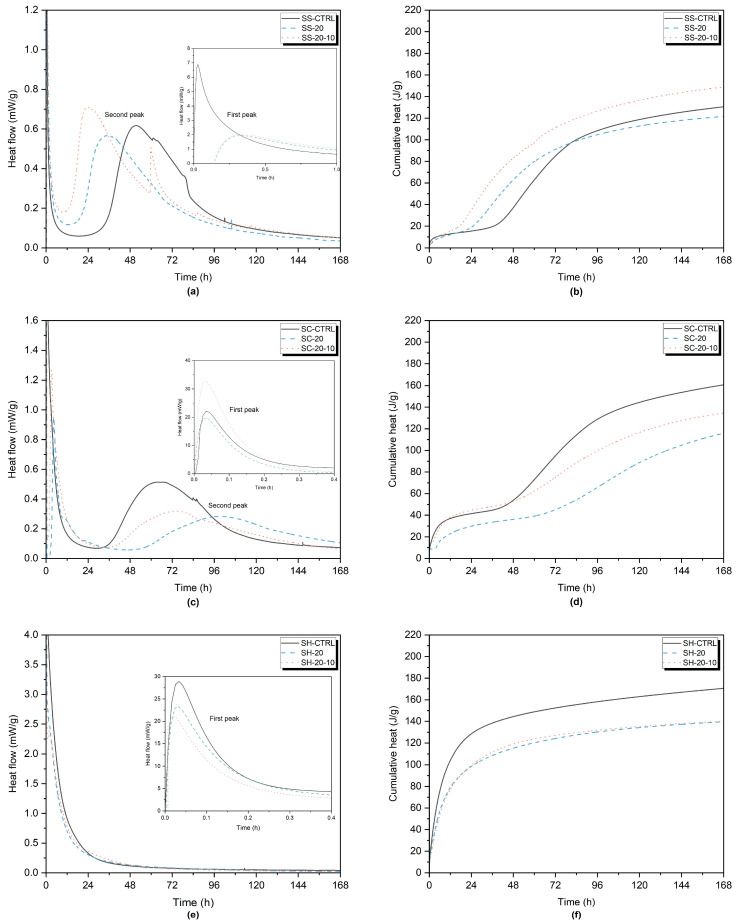
Heat flow of (**a**) SS-activated pastes, (**c**) SC-activated pastes, (**e**) SH-activated pastes; Cumulative heat of (**b**) SS-activated pastes, (**d**) SC-activated pastes, (**f**) SH-activated pastes.

The hydration process, which is also present in geopolymers based on GGBFS can be divided into five stages: dissolution, induction, acceleration, deceleration, and slow down period [103,104]. The heat flow and cumulative heat of the selected SS-activated samples are provided in Figure 11a,b, respectively. The first peak of the control sample, SS-CTRL, was significantly higher than that of the SS-20 and SS-20-10 samples. This observation aligns with the findings of Dai et al. [91], who reported similar heat flow results for hybrid mixes of GGBFS and fly ash. They proposed that this could be attributed to a higher degree of depolymerization of GGBFS compared to fly ash.

On the other hand, the first peaks of wood ash-containing samples, regardless of the grinding process, showed similar results. This suggests that the grinding of wood ash did not significantly impact its dissolution rate of GGBFS. The second peaks, corresponding to the formation of the main reaction products, were observed between 24 and 51 h. The second peak of the SS-20-10 sample occurred earlier and with higher intensity compared to the SS-CTRL and SS-20 samples. The incorporation of wood ash, particularly the ground wood ash, led to a shortened induction stage. Moreover, the cumulative heat release of the SS-20-10 sample was the highest at 168 h. The cumulative heat release for the SS-20-10 sample was 148.5 J/g, slightly higher than the control sample, SS-CTRL after 7 days. A third peak, which is rare in SS-activated materials, was observed in SS-20-10, which might also increase the cumulative heat. Bílek et al. [105] explained this peak formation as a gelation of activator or primary C-A-S-H formation. Conversely, the SS-20 sample exhibited a cumulative heat release of 121.3 J/g, indicating a slightly lower value than the control sample. The positive effect of grinding on mechanical properties might be supported by these findings. Moreover, it might be explained by possible nucleation sites that might be created by wood ash, as discussed in Section 3.2.

The induction period of SC-activated samples was slightly delayed compared to SS and SH-activated samples [86]. For the SC-activated samples, the second hydration peak of the slag was at a lower intensity and delayed significantly, as reported by [15]. The period of hydration induction was longer [86]. The second peaks of the SC-activated samples were observed between 64.5 and 100 h. The wood ash-containing samples showed lower intensity peaks and slow hydration processes. SC-20-10 released the highest cumulative heat in the first 44 h but then SC-CTRL released the highest cumulative heat, which was 160.5 J/g at 168 h. The first peak of SC-20-10 also exhibited a significantly higher peak compared to SC-CTRL and SC-20.

For the SH-activated samples, the second peak did not occur, possibly due to the low formation of reaction products within the paste during the 7-day period. This might be supported by the low intensity of the peak corresponding to C-A-S-H in the XRD pattern.

The pH value is an important factor for hydration and strength. NaOH has higher pH compared to Na_2_SiO_3_ and it usually may cause a faster dissolving of GGBFS, shorten the induction period and accelerate the early hydration step [86]. However, it also reduces the strength [106]. The selected SH-activated and SS-activated samples demonstrated comparable induction periods. This might be explained by their pH values being in close proximity to each other. However, the cumulative heat of SH-activated samples was higher compared to the SS-activated samples during the hydration process, except for the sample SS-20-10. The cumulative heat of the SH-20 and SH-20-10 was 140 J/g and SH-CTRL released the highest heat, which was 170.5 J/g. Similar findings were reported in the literature [107].

## 4. Conclusions

In this study, the strength and microstructure properties of alkali-activated mortars containing different levels (0 wt%, 10 wt%, and 20 wt%) of partial GGBS replacement of ground wood ash at two different durations and using three different alkali activators were compared.

- It has been observed that the grinding of wood ash reduces the particle size and has a positive effect on the mechanical strength, and the optimum grinding time is 10 min.
- The SS-activated mortars had the highest compressive strength values. Moreover, SS-activated 10 wt% 10 min ground wood ash improved the compressive strength compared to the control sample.
- The flexural strength results showed similar trend as the compressive strength. However, SH-activated samples exhibited unexpectedly high results which needs further investigation.
- Increasing the wood ash ratio increased the porosity for all alkali-activator types. Of these, the microstructure of SS-activated mortars had the most homogeneous and denser structure while SC-activated mortars had the most porous.
- The XRD results confirmed that the formation of C-A-S-H was the main reaction product for all the samples.

The results suggest that wood ash is a promising material for using in alkali-activated systems and ball milling has a positive effect on the mechanical properties compared to untreated wood ash.

## Figures and Tables

**Table 1:** Chemical composition of non-treated WA, WA10 and WA20, and GGBFS.

Chemical Composition (%)	WA	WA10	WA20	GGBFS
SiO_2_	22.40	22.30	21.70	34.80
Al_2_O_3_	6.75	6.87	6.77	11.30
Fe_2_O_3_	2.62	3.11	3.39	0.42
CaO	15.10	16.00	15.60	40.80
K_2_O	8.25	8.96	9.27	0.99
MgO	2.69	2.84	2.84	11.40
MnO	0.80	0.85	0.82	0.32
P_2_O_5_	2.81	2.96	2.97	<0.02
TiO_2_	0.30	0.30	0.28	1.46
Na_2_O	1.46	1.48	1.47	0.58
LOI (1000 °C)	29.70	32.30	31.30	−1.81
Alkali content (K_2_O + Na_2_O)	9.71	10.44	10.74	1.57
pH	10.48	10.62	10.78	10.38

**Table 2:** Alkali-activated mortar mix design.

Mortar Mix ID	Wood Ash Grinding Time (min)	Slag:Wood Ash Ratio	Alkali Activator Type	Alkali Activator Dosage	Alkali Modulus (Ms)	pH of Alkali Solution
SS-CTRL	-	100:0	SS	10%	1	12.84
SS-10	0	90:10	SS	10%	1	12.84
SS-20	0	80:20	SS	10%	1	12.84
SS-10-10	10	90:10	SS	10%	1	12.84
SS-20-10	10	80:20	SS	10%	1	12.84
SS-10-20	20	90:10	SS	10%	1	12.84
SS-20-20	20	80:20	SS	10%	1	12.84
SC-CTRL	-	100:0	SC	10%	-	11.24
SC-10	0	90:10	SC	10%	-	11.24
SC-20	0	80:20	SC	10%	-	11.24
SC-10-10	10	90:10	SC	10%	-	11.24
SC-20-10	10	80:20	SC	10%	-	11.24
SC-10-20	20	90:10	SC	10%	-	11.24
SC-20-20	20	80:20	SC	10%	-	11.24
SH-CTRL	-	100:0	SH	10 M	-	12.95
SC-10	0	90:10	SH	10 M	-	12.95
SC-20	0	80:20	SH	10 M	-	12.95
SH-10-10	10	90:10	SH	10 M	-	12.95
SH-20-10	10	80:20	SH	10 M	-	12.95
SH-10-20	20	90:10	SH	10 M	-	12.95
SH-20-20	20	80:20	SH	10 M	-	12.95

SS: Sodium silicate, SC: Sodium carbonate, SH: Sodium hydroxide.

**Table 3:** Calculated average atomic ratios based on results obtained from EDS spot analysis results for alkali-activated mortars.

Sample	Ca/Si	Ca/Al	Al/Si	Na/Si
SS-CTRL	0.71	4.15	0.18	0.42
SS-10	0.65	3.72	0.18	0.67
SS-20	0.72	3.55	0.21	0.56
SS-10-10	0.79	3.79	0.22	0.57
SS-20-10	0.75	3.70	0.22	0.57
SS-10-20	0.90	3.42	0.29	0.45
SS-20-20	0.75	3.37	0.24	0.49
SC-CTRL	0.81	3.12	0.29	0.45
SC-10	0.72	3.19	0.24	0.69
SC-20	0.71	3.16	0.24	0.57
SC-10-10	1.05	3.94	0.30	0.67
SC-20-10	1.22	3.70	0.35	1.07
SC-10-20	0.75	3.37	0.24	0.48
SC-20-20	0.91	3.01	0.37	0.67
SH-CTRL	0.63	3.80	0.17	1.89
SH-10	0.55	3.40	0.14	3.53
SH-20	0.70	3.69	0.20	3.79
SH-10-10	0.98	3.94	0.26	1.15
SH-20-10	0.71	3.51	0.22	1.36
SH-10-20	0.81	4.04	0.21	1.60
SH-20-20	0.89	3.65	0.25	1.25

## Data Availability

Available upon request.
